# Induction of Micronuclei in Cervical Cancer Treated with Radiotherapy

**DOI:** 10.3390/jpm10030110

**Published:** 2020-09-03

**Authors:** Daijiro Kobayashi, Takahiro Oike, Kazutoshi Murata, Daisuke Irie, Yuka Hirota, Hiro Sato, Atsushi Shibata, Tatsuya Ohno

**Affiliations:** 1 Department of Radiation Oncology, Gunma Prefectural Cancer Center, Gunma 373-8550, Japan; m07201029@gmail.com; 2 Department of Radiation Oncology, Gunma University Graduate School of Medicine, Gunma 371-8511, Japan; daisuke_i@gunma-u.ac.jp (D.I.); yukahirota@gunma-u.ac.jp (Y.H.); hiro.sato@gunma-u.ac.jp (H.S.); tohno@gunma-u.ac.jp (T.O.); 3 QST Hospital, National Institute for Quantum and Radiological Science and Technology, Chiba 263-8555, Japan; murata.kazutoshi@qst.go.jp; 4 Gunma University Initiative for Advanced Research (GIAR), Gunma University, Gunma 371-8511, Japan; shibata.at@gunma-u.ac.jp; 5 Gunma University Heavy Ion Medical Center, Gunma 371-8511, Japan

**Keywords:** micronuclei, uterine cervical cancer, radiotherapy, cGAS, STING, abscopal effect, immunotherapy

## Abstract

Micronuclei (MN) trigger antitumor immune responses via the cyclic GMP-AMP synthase-signaling effector stimulator of interferon genes (cGAS-STING) pathway. Radiotherapy induces MN in peripheral blood lymphocytes. However, data for solid tumors are lacking. Here, we analyzed MN post-radiotherapy in solid tumor samples. Tumor biopsy specimens were obtained from seven prospectively recruited patients with cervical cancer, before treatment and after receiving radiotherapy at a dose of 10 Gy (in five fractions). The samples were stained with 4′,6-diamidino-2-phenylindole dihydrochloride, and 200 nuclei per sample were randomly identified and assessed for the presence of MN or apoptosis, based on nuclear morphology. The median number of MN-harboring nuclei was significantly greater in samples from patients treated with radiotherapy than in pre-treatment samples (151 (range, 16–327) versus 28 (range, 0–61); *p* = 0.015). No significant differences in the number of apoptotic nuclei were observed between pre-treatment and 10 Gy samples (5 (range, 0–30) versus 12 (range, 2–30); *p* = 0.30). This is the first report to demonstrate MN induction by radiotherapy in solid tumors. The results provide clinical evidence of the activation of antitumor immune responses by radiotherapy.

## 1. Introduction

Immunotherapy is rapidly becoming a promising strategy for cancer treatment. Recent reports on the combination use of immunotherapy with radiotherapy (RT) are overwhelming [1,2]. However, this kind of treatment strategy is hampered by the fact that its efficacy is unpredictable [1]. The cost for cancer immunotherapy is unsustainably high [1], therefore, a stratification of the patients who benefit from the combination treatment is needed. To this end, at the present, identification of reliable biomarkers of response to cancer immunotherapy combined with radiotherapy represents one of the mainstream immune-oncology research lines [1].

Micronuclei (MN) are induced following aberrant mitotic events in response to ionizing radiation, and are identified as one or a few smaller nuclei independent from the main nucleus [3,4]. After irradiation, cyclic GMP-AMP (cGAMP) synthase (cGAS) localizes to MN and binds to double-stranded DNAs (dsDNAs) that activate the stimulator of interferon genes (STING), an endoplasmic reticulum adaptor protein [5,6]. This induces GAMP-driven proinflammatory response [7,8], leading to type-1 interferon secretion [9]. As such, MN formation is directly related to the activation of cGAS-STING pathway that is the key axis of antitumor immune responses following radiation [7,8,10,11].

Taken together, RT-induced intratumoral MN may be used as a biomarker of the response to cancer immunotherapy combined with radiotherapy. Induction of MN by RT has been demonstrated in peripheral blood lymphocytes obtained from individuals treated with RT [12,13,14,15]. However, the induction of MN by RT in solid tumors has not been reported. This study aimed to obtain proof of principle for the induction of MN by RT in solid tumors. To this end, we evaluated tumor specimens obtained at pre-treatment and intra-RT time points from the same patients with cervical cancer.

## 2. Materials and Methods

### 2.1. Patients

Patients with cervical cancer were prospectively enrolled in the study if they met the following criteria: (a) pathologically-confirmed newly diagnosed cervical cancer; (b) treated with definitive RT at Gunma University Hospital between November 2017 and November 2018; (c) staged as IB1–IVA according to the 2009 International Federation of Gynecology and Obstetrics staging system; and (d) no previous exposure to radiotherapy or cytotoxic agents.

This study was approved by the Ethical Review Board Committee of Gunma University Hospital (approval number, 1109). Written consent was obtained from all participants.

### 2.2. Treatment

Patients were treated with concurrent chemoradiotherapy using standardized protocols [16]. Briefly, RT consisted of EBRT using 10 MV X-rays and computed tomography-based high dose-rate image-guided brachytherapy (IGBT). For EBRT, whole pelvic irradiation was delivered at a dose of 50 Gy in 25 fractions, and a central shielding technique was used for the latter 10–30 Gy. IGBT was performed with a ^192^Ir Remote-After-Loading System (microSelectron, Elekta, Stockholm, Sweden), using tandem and ovoid applicators. A total of 24 Gy was delivered in four fractions, one fraction per week. Cisplatin (40 mg/m^2^) was administered weekly during the RT treatment period, with the first course administered on day 1. Patients ≥ 75 years old did not receive chemotherapy.

### 2.3. Sample Collection and Nuclear Morphology Assessment

Specimens were obtained by punch biopsy from the center of tumors of the uterine cervix before the initiation of RT and after RT at 10 Gy. The nuclei contained in the tumor specimens were stained with DAPI (Sigma-Aldrich, St. Louis, MO, USA), as described previously [17]. Briefly, the biopsy specimens were kept in ice-cold phosphate buffered saline (PBS) and transported immediately from the hospital to the laboratory. Within 10 min from the biopsy, the tumor was minced using a surgical knife and incubated in a solution containing 0.25% trypsin (Thermo Scientific, Waltham, MA, USA) and 1 mM ethylenediaminetetraacetic acid (Sigma-Aldrich) at 37 °C for 5 min. The samples were then passed through a 70-micron nylon mesh cell strainer (Falcon, New York, NY, USA) in 5 mL PBS. Strained cells were collected by centrifugation at 1500× *g* rpm for 5 min and suspended in 0.4 mL PBS. A Cytospin™ 4 Cytocentrifuge (Thermo Scientific) was used to spread 0.1 mL of the cell suspension in a monolayer on slide glasses. The cells were then fixed with 3% paraformaldehyde-2% sucrose solution for 10 min. Fixed cells were incubated with 0.7% Triton X-100 (Sigma-Aldrich) and stained with DAPI. Coverslips were mounted in Vectashield (Vector Laboratories, Burlingame, CA, USA).

**Figure 1:**
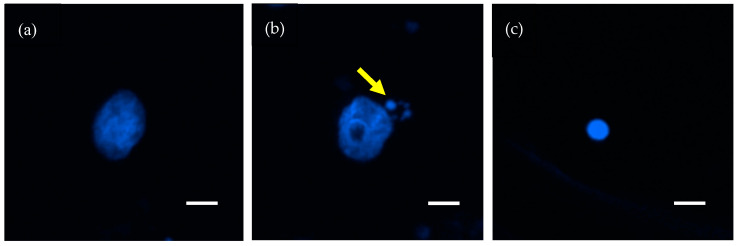
Representative images of nuclei stained with 4′,6-diamidino-2-phenylindole dihydrochloride. (**a**) Normal nucleus. (**b**) Nucleus harboring micronuclei (arrow). (**c**) Apoptosis. Scale bar = 10 μm.

Microscopic images were obtained under a fluorescence microscope (Eclipse Ni, Nikon, Tokyo, Japan), using the settings for conventional image capture with a 40× objective lens. In continuous fields, 200 nuclei were identified and the nuclear events (i.e., MN and apoptosis) for each nucleus were recorded. MN were determined by the presence of small fragments separate from the main nuclei (Figure 1b) [8]. Apoptosis was determined by the presence of apoptotic bodies, nuclear condensation, or nuclear fragmentation (Figure 1c) [18].

### 2.4. Statistical Analysis

Differences in the prevalence of nuclear events between pre-RT and post-RT samples in the same patient were examined by paired non-parametric Wilcoxon test. Differences in the induction rate of nuclear events between the CCRT group and the RT group alone were examined by unpaired non-parametric Mann–Whitney test. Correlations of the interval between two biopsies with the induction rate of nuclear events were examined by Spearman’s rank order test. Differences were considered statistically significant at *p* < 0.05. Statistical analysis was performed using Prism8 (GraphPad, San Diego, CA, USA).

## 3. Results

**Figure 2:**
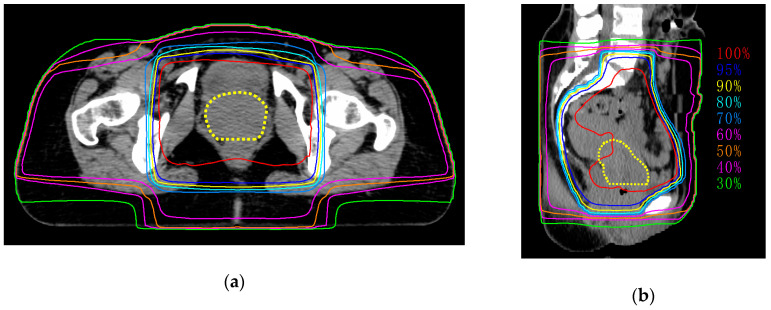
Representative images of dose distribution of external beam radiotherapy. Dashed line indicates tumor (yellow). (**a**) Axial plane; (**b**) sagittal plane.

Seven patients were enrolled in this study (Table 1). The median age was 57 (42–82) years. Four patients received concurrent chemoradiotherapy (CCRT) with weekly cisplatin (40 mg/m^2^), and the remaining three patients received RT alone because of older age (i.e., ≥ 75 years). The median total dose of external beam radiotherapy (EBRT) was 55.6 (50.0–58.0) Gy. We confirmed that more than 95% of the planned doses were appropriately delivered to tumors (Figure 2).

Tumor biopsy was performed before the initiation of RT and after exposure to 10 Gy in the same patients. The median interval between first biopsy and second biopsy was 7 days (range, 4–10 days). Two patients with a 4-day interval underwent biopsies on Monday and Friday of the same week. The patient with a 10-day interval underwent biopsy before and after the year-end holiday. The other four patients underwent biopsies on Monday of the first week and Monday or Tuesday of the following week (i.e., 7- and 8-day intervals, respectively).

MN and apoptosis in each sample were determined by morphological assessment of nuclei stained with 4′,6-diamidino-2-phenylindole, dihydrochloride (DAPI) (see Section 2.3 for detailed methods). Representative images of MN and apoptosis are shown in Figure 1. 

**Figure 3:**
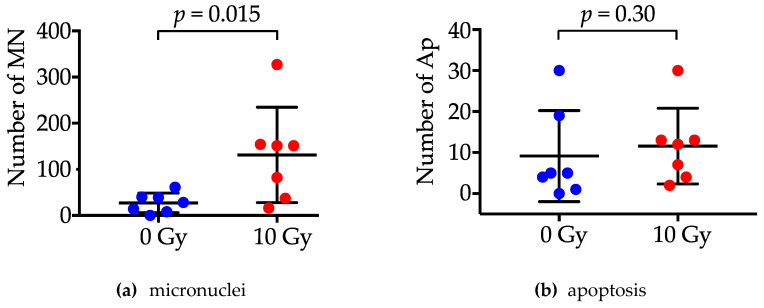
The number of nuclei harboring micronuclei (MN) (**a**) and those undergoing apoptosis (Ap) (**b**) pre-radiotherapy or after 10 Gy RT. *p* values were assessed by the paired non-parametric Wilcoxon test.

A random 200 nuclei were evaluated for each experimental setting; the median number of nuclei harboring MN was 28 (range, 0–61) pre-RT and 151 (range, 16–327) after RT, at a dose of 10 Gy. The number of MN-harboring nuclei was significantly greater in 10 Gy samples than in pre-RT samples (*p* = 0.015) (Figure 3). In contrast to MN, the number of apoptotic nuclei did not differ significantly between the pre-RT samples and the 10 Gy samples (5 (range, 0–30) versus 12 (range, 2–30); *p* = 0.30).

**Figure 4:**
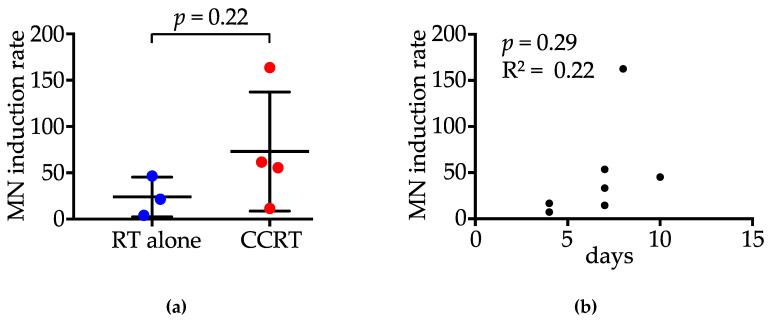
Induction rate of micronuclei (MN) at 10 Gy, relative to pre-RT settings. (**a**) Comparison between patients treated with radiotherapy (RT) alone and those receiving concurrent chemoradiotherapy (CCRT). *p* value was assessed by the Mann–Whitney test. (**b**) Correlation between the interval between two biopsies and the induction rate of MN. *p* value and R square value were assessed by Spearman’s Rank Order test.

To analyze the influence of chemotherapy administered concurrently with RT on MN induction, we compared the rate of MN-harboring nuclei at 10 Gy relative to that for pre-RT settings (i.e., MN induction rate) between the CCRT group (*n* = 4) and the RT alone group (*n* = 3) (Figure 4a). The median MN induction rate was 21.5% (4.0–46.5%) in patients treated with RT alone and 58.5% (11.5–163.5%) in those treated with CCRT; there was no significant difference in MN induction rate between the two groups (*p* = 0.22). The interval between the first and second biopsies was not significantly correlated with the MN induction rate (*p* = 0.29, R^2^ = 0.22) (Figure 4b).

## 4. Discussion

This study examined the induction of MN by radiotherapy, using cervical biopsy samples obtained before and after radiotherapy. Intratumoral MN showed a 5.4-fold increase after RT for 10 Gy in 5 fractions compared with pre-treatment setting. The increase in MN after irradiation was comparable with that reported previously in peripheral blood lymphocytes [12,13,14,15]. The novelty of this study is that, for the first time, we demonstrated the induction of MN in primary tumors treated with clinical radiation beams. In contrast to MN, there was no significant difference in the rate of apoptosis before and after irradiation. In a previous in vitro study, we investigated the nuclear morphology of cell death after X-ray irradiation at 4 Gy [19]. In that study, the most common mode of clonogenic cell death induced by X-rays was mitotic catastrophe, and the median rate of apoptosis was approximately 1%. MN represent an increase in genetic instability, and they are a hallmark of mitotic catastrophe [20,21,22], which is consistent with the results of this study. In addition, it is worth noting that a previous study showed that excessive expression of Trex1 by high dose irradiation in a single fraction decreases cytosolic dsDNA [11]. These data indicate that the fractionated RT schedule employed in the present study (i.e., 2 Gy per fraction and five fractions per week) might be effective in inducing MN.

Advances in radiotherapy techniques have improved the outcomes of patients with newly diagnosed uterine cervical cancer [16]. However, the platinum-based combinations used in first-line chemotherapy for metastatic or recurrent tumors rarely provide durable disease control [23]. McLachlan et al. reported the outcome of second-line chemotherapy in the patients with recurrent or metastatic cervical cancer [24]. In that report, they showed the response rate was insufficient as the median overall survival was 9.3 months. As such, better treatments are still needed for patients with recurrent or metastatic cervical cancer, and patients should be considered for clinical trials whenever feasible, including novel targeted agents and immunotherapy.

Almost all cases of uterine cervical cancer are caused by high-risk human papillomavirus (HPV) infection [25] and HPV expresses foreign antigens within host cells. Although immunotherapy has the potential to improve the survival of uterine cervical cancer patients, the response rate of uterine cervical cancer to checkpoint immunotherapy is 10–25% [26]. Previous studies suggest the differences in the immunological microenvironments and escape mechanisms by histological subtypes. Reddy et al. showed that the expression of programmed cell death-ligand 1 (PD-L1) was higher in squamous cell carcinoma than in adenocarcinoma [27]. Boussios et al. showed that diffuse PD-L1 expression in squamous cell carcinoma is correlated with poor disease-free survival and disease-specific survival compared to the cases with marginal PD-L1 expression; in adenocarcinoma, survival benefit was observed for the patients with tumors lacking PD-L1-positive tumor-associated macrophages [28,29]. The cohort of the present study solely consisted of squamous cell carcinoma. From these perspectives, future studies should compare post-RT MN induction between squamous cell carcinoma and adenocarcinoma.

The abscopal effect is an anti-tumor effect observed in the unirradiated sites of RT-treated patients. Abscopal effect “comes by chance, not through seeking”, i.e., not observed in all RT-treated cases and the methods for its prediction has not been established, despite a number of clinical trials as summarized by Wang and colleagues [30]. This heterogeneity in the induction of abscopal effect post-RT can be attributable to the variance in the strength of post-RT antitumor immune responses. cGAS-STING pathway may be the mediator of post-RT abscopal effect, and MN may be the predictive biomarker. This possibility warrants further investigation in future.

In this study, we selected the time point corresponding to a dose of 10 Gy irradiation. Gamulin et al. reported that the number of MN increases gradually during RT, reaching the highest value after the administration of the last RT fraction [31]. Nevertheless, we thought, from clinical experience of rapid shrinkage of uterine cervical tumors during RT course, that sufficient samples of viable cells may not be available at later time points; therefore, we chose 10 Gy for sample collection in this study.

## 5. Conclusions

MN trigger antitumor immune responses via the cGAS-STING pathway. For the first time, we present clinical evidence of MN induction by RT in solid tumors. The present data provide an important insight into the activation of antitumor immune responses by RT.

## Figures and Tables

**Table 1:** Patient characteristics.

Characteristics	Median (Range)
Age (years)	57 (42–82)
Age ≤ 50 vs. > 50	3 vs. 4
Menstrual status (premenopausal vs. postmenopausal)	2 vs. 5
Follow-up (months)	11 (1–21)
FIGO stage	
IB	0
II	4
III	2
IVA	1
Histology	
Squamous cell carcinoma	7
Tumor diameter (cm)	61.3 (41.1–80.1)
Serum tumor marker	
CEA (ng/mL)	4.4 (0.9–7.4)
SCC (ng/mL)	8.9 (0.9–28.3)
Treatment method	
Chemoradiotherapy	4
Radiotherapy alone	3
EBRT dose	55.6 Gy/29 fr.
IGBT dose	24 Gy/4 fr.
Interval between first and second biopsy (days)	7 (4–10)
Response at the end of EBRT (absence of gross residual tumor vs. presence of gross residual tumor)	1 vs. 6

FIGO, International Federation of Gynecology and Obstetrics; CEA, carcinoembryonic antigen; SCC, squamous cell carcinoma antigen; EBRT, external beam radiotherapy; IGBT, image-guided brachytherapy; fr., fraction.

## Abbreviations

CCRT	Concurrent chemoradiotherapy
cGAS	Cyclic GMP-AMP synthase
DAPI	4′,6-diamidino-2-phenylindole, dihydrochloride
EBRT	External beam radiotherapy
IGBT	Image-guided brachytherapy
IMRT	Intensity modulated radiotherapy
MN	Micronuclei
PBS	Phosphate buffer saline
PD-L1	Programmed cell death-ligand 1
RT	Radiotherapy
STING	Stimulator of interferon genes
